# Cytotoxic T lymphocyte antigen 4 expression in human breast cancer: implications for prognosis

**DOI:** 10.1007/s00262-015-1696-2

**Published:** 2015-04-17

**Authors:** Haiming Yu, Junlan Yang, Shunchang Jiao, Ying Li, Wei Zhang, Jiandong Wang

**Affiliations:** 1 Department of Medical Oncology, General Hospital of Chinese People’s Liberation Army, No. 28, Fuxing Road, Haidian District, Beijing, 100853 People’s Republic of China; 2 Department of Pathology, 401 Hospital of People’s Liberation Army, Qingdao, People’s Republic of China; 3 Department of General Surgery, General Hospital of Chinese People’s Liberation Army, Beijing, People’s Republic of China

**Keywords:** Breast cancer, Disease-free survival, Overall survival, Cytotoxic T lymphocyte antigen 4, CTLA-4

## Abstract

To examine the relationship between cytotoxic T lymphocyte antigen 4 (CTLA-4) expression and breast cancer prognosis, CTLA-4 expression was immunohistochemically detected in paraffin-embedded specimens of primary tumors from 130 patients with breast cancer who had a mean follow-up period of 112 months. CTLA-4 expressed in cytoplasm of breast cancer cells and in cytoplasm and cell membranes of interstitial lymphocytes. Univariate analysis (log-rank) associated higher density of interstitial CTLA-4^+^ lymphocytes with longer DFS and OS, but higher tumor CTLA-4 expression with shorter OS. After controlling for age, clinical stage, Scarff-Bloom-Richardson grade, tumor thrombus, ER, PR, HER2 and Ki-67, multivariate analysis (Cox) showed that density of interstitial CTLA-4^+^ lymphocytes independently predicted longer DFS (HR 0.315, *P* = 0.002) and OS (HR 0.313, *P* = 0.005), whereas tumor CTLA-4 expression independently predicted shorter DFS (HR 2.176, *P* = 0.029) and OS (HR 2.820, *P* = 0.007), i.e., patients with high CTLA-4^+^ lymphocyte density and CTLA-4^low^ tumor cells had the best prognoses. These results indicated that CTLA-4 expression in lymphocytes was associated with better prognosis, but that in tumor cells was associated with worse prognosis. Patients’ CTLA-4 profiles might thus be used to predict the benefits and toxicity of CTLA-4 blockade.

**Electronic supplementary material:**

The online version of this article (doi:10.1007/s00262-015-1696-2) contains supplementary material, which is available to authorized users.

## Introduction

Tumor-derived immune dysregulation is a key feature of breast cancer. The immunosuppressive microenvironment derived from breast cancer cells consists of cytokines and immune checkpoint molecules that can block anti-tumor immunity [1–3]. One of these immune checkpoint molecules is cytotoxic T lymphocyte antigen 4 (CTLA-4, CD152). CTLA-4 is a CD28 homologue and shares two ligands—B7-1 (CD80) and B7-2 (CD86)—with CD28. CTLA-4 has much stronger binding affinity for the two ligands than CD28 [4]. CTLA-4 has three different isoforms: the full-length isoform with an extracellular ligand-binding domain and an intracellular signal-transducing domain; the soluble isoform that consists only of the extracellular domain; and the third isoform (which has only been identified in mice), which lacks the extracellular domain [5].

CTLA-4 is normally expressed at low levels on the surface of naive effector T cells and regulatory T cells (Tregs). After stimulation of a naive T cell through the T-cell receptor, CD8^+^ T cells and CD4^+^ T cells, including Tregs, up-regulate membrane CTLA-4 and secrete soluble CTLA-4 [6–8]. As negative feedback to maintain immune self-tolerance and homeostasis, different CTLA-4 isoforms reduce T-cell activation through either intrinsic or extrinsic regulation of T-cell activity. When CD28 binds B7 receptors on antigen-presenting cells and mediates activating signals in T cells, the full-length form of CTLA-4 binds B7 and initiates inhibitory signals via its intracellular signal-transducing domain, including cell-cycle arrest and decreased cytokine production. Upon T-cell activation, intracellular calcium levels are elevated, and secretary granules containing presynthesized soluble CTLA-4 are translocated to the central supramolecular activation cluster (cSMAC) within the immunological synapse to release the soluble CTLA-4. Soluble CTLA-4 interacts with B7, which excludes CD28 from the cSMAC [9]. Ex vivo experiments showed soluble CTLA-4 to inhibit human T-cell responses to antigen; blocking soluble CTLA-4 significantly enhanced antigen-driven PBMC (peripheral Blood Mononuclear cell) responses [8].

Previous studies implicated CTLA-4 in immune dysregulation of breast cancer and found CTLA-4 to be highly expressed in breast tumor cells [10, 11]. Plasma soluble CTLA-4 and CTLA-4 expression in peripheral mononuclear cells of breast cancer patients were higher than in normal controls [10, 12, 13]. Nonetheless, the relationship between prognosis and CTLA-4 expression in breast cancer remains elusive.

## Materials and methods

### Patients

This retrospective study included 130 patients who underwent breast cancer surgery between January 2000 and December 2002 at the People’s Liberation Army General Hospital, Beijing, China. The study was approved by the Institutional Review Board of the People’s Liberation Army General Hospital. Informed consents were obtained from all the patients. Inclusion criteria include: (1) pathologically confirmed breast cancer, (2) availability of paraffin-embedded specimens of the primary tumor and (3) relatively complete follow-up data. Of 175 consecutive patients who underwent radical mastectomies, we excluded 32 patients whose primary tumor specimens were unavailable and 13 whose follow-up data were unavailable. Finally, 130 patients were included.

### Immunohistochemistry

Serial paraffin-embedded sections (3 μm thick) from the 130 patients were de-waxed with xylene and subsequently hydrated with an ethanol gradient. The tissue sections were subjected to high-pressure Tris–EDTA buffer (pH 9.0) for antigen retrieval and then immersed in 3 % H_2_O_2_ for 10 min to eliminate endogenous peroxidase activity. A working solution of normal goat serum was added to the tissue sections and incubated at 37 °C in a humidified box for 10 min to block nonspecific antigens. Sections were then incubated overnight at 4 °C with rabbit antihuman CTLA-4 IgG (1:100 dilution, bs-1179R, Beijing Biosynthesis Biotechnology Co., Ltd, China) and then at 37 °C for 30 min with a secondary antibody against rabbit and mouse immunoglobulins (ready-to-use, EnVision K500711, Dako, Denmark). Afterward, sections were stained with DAB for 1 min, and nuclei were counterstained with hematoxylin. Slides were dehydrated in an ethanol gradient, mounted with neutral gum and stored for later observation. Sections of human tonsil specimens with confirmed high expression of the target molecules served as positive control. Sections incubated with the primary antibody diluent with no antibody were used as negative control 1; sections incubated with primary antibody (1:100 dilution, bs-1179R, Beijing Biosynthesis Biotechnology) premixed with CTLA-4 peptide (bs-1179P, Beijing Biosynthesis Biotechnology) were used as negative control 2 (Supplementary Figure 1).

### Imaging and data analysis

IHC slides were evaluated by two independent pathologists who were unaware of patients’ clinical and prognostic information. Two variables—density of interstitial CTLA-4^+^ lymphocytes and tumor CTLA-4^+^ expression—were evaluated as follows: interstitial CTLA-4^+^ cells in interstitial areas adjacent to tumor nests were counted in 15 high-power fields adjacent to tumor nests (400×) that were randomly selected from the entire film under a Leica DM2000 microscope. Interstitial CTLA-4^+^ cells per high-power field were counted; density of interstitial CTLA-4^+^ lymphocytes (average number of CTLA-4^+^ cells per mm^2^) was calculated by dividing positive cells by the area of high-power fields (0.31 mm^2^). CTLA-4 expression in tumor cell cytoplasm was semiquantitatively scored based on staining intensity [14] (none: 0; weak: 1; moderate: 2; and strong: 3; examples in Supplementary Figure 2). Percentages of staining intensities in tumor cell cytoplasm were sequentially calculated as A, B, and C %. Tumor CTLA-4 expression (CTLA-4 intensity in tumor cell cytoplasm) was determined as (1 × A % + 2 × B % + 3 × C %).

### Statistical analysis

Statistical analyses were performed using the SPSS 13.0 statistical software package. Correlations between continuous variables were assessed using the Spearman rank-sum test. Correlations between categorical variables and clinicopathological parameters were evaluated by employing Mann–Whitney U test or Kruskal–Wallis test. Nonparametric receiver-operating characteristic (ROC) analysis was used to determine optimal cutoff values of variables for overall survival (OS). Patients were divided into high- and low-expression groups in terms of optimal cutoff values of tumor CTLA-4 expression (CTLA-4^high^ and CTLA-4^low^) or density of interstitial CTLA-4^+^ lymphocytes (density^high^ and density^low^). These groups were subjected to univariate and multivariate survival analyses. For survival analysis, the Kaplan–Meier method was used. For univariate analysis of significance, the log-rank test or Cox analysis was used. The Cox proportional hazards model was used for multivariate analysis. *P* < 0.05 was considered as statistically significant.

With the actual OS as the gold standard, sensitivity and specificity for factors to predict survivals were determined by crosstabs. Predictive effects were determined by levels of sensitivity, specificity, Youden index and accuracy.

### Patient characteristics

Clinicopathological data of enrolled subjects were summarized in Table 1. No patient received surgical castration, neo-adjuvant chemotherapy or targeted therapy. Median follow-up was 112 months (range 7.7–138.6 months). Of 33 patients who suffered local recurrence or metastasis, 29 patients died. Disease-free survival (DFS) and OS rates were 74.6 and 77.7 %, respectively. Median DFS and OS were not obtained.

**Table 1:** Clinicopathological features of the 130 patients with breast cancer

Clinicopathological parameter	*N* (%)
Age (years)	
≤48	66 (50.8)
>48	64 (49.2)
Menstrual status	
Premenopausal	82 (63.1)
Postmenopausal	48 (36.9)
Pathological type	
Invasive ductal carcinoma	120 (92.3)
Invasive lobular carcinoma	2 (1.5)
Medullary carcinoma	3 (2.3)
Mucinous carcinoma	2 (1.5)
Invasive eczematous carcinoma of nippla	2 (1.5)
Invasive ductal carcinoma and invasive lobular carcinoma	1 (0.8)
Tumor size	
T1	47 (36.2)
T2	70 (53.8)
T3	11 (8.5)
T4	2 (1.5)
Lymph node metastasis	
N0	63 (48.5)
N1	39 (30.0)
N2	13 (10.0)
N3	15 (11.5)
Clinical stage	
I	31 (23.8)
IIA	42 (32.3)
IIB	24 (18.5)
IIIA	16 (12.3)
IIIB	2 (1.5)
IIIC	15 (11.5)
SBR grading	
I	17 (13.1)
II	89 (68.5)
III	24 (18.5)
Thrombosis	
Positive	30 (23.1)
Negative	100 (76.9)
ER	
Positive	84 (64.6)
Negative	46 (35.4)
PR	
Positive	74 (56.9)
Negative	56 (43.1)
HER-2	
Positive	27 (20.8)
Negative	103 (79.2)
Adjuvant chemotherapy	
Yes	104 (80.0)
No	26 (20.0)
Adjuvant radiotherapy	
Yes	80 (61.5)
No	50 (38.5)
Adjuvant endocrine therapy	
Yes	71 (54.6)
No	59 (45.4)

### CTLA-4 expression in breast cancer

**Fig. 1:**
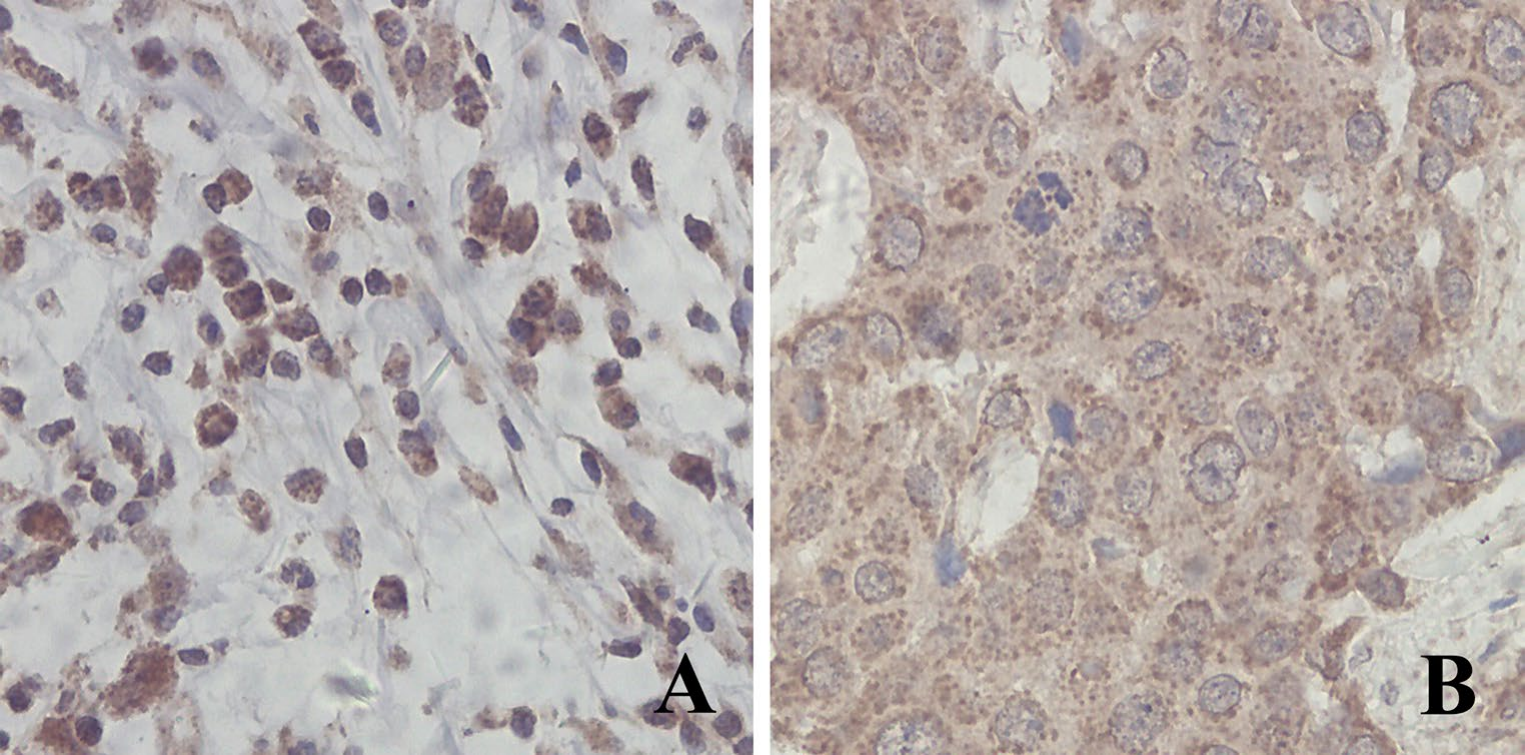
Expression profiles of CTLA-4 in breast cancer. CTLA-4 was expressed in cytoplasm and cell membrane of interstitial lymphocytes (**a**); CTLA-4^+^ cytoplasmic dots were found in cytoplasm of tumor cells (**b**)

CTLA-4 was expressed in cytoplasm and cell membrane of interstitial lymphocytes (Fig. 1a); CTLA-4^+^ discrete cytoplasmic dots were found in cytoplasm of tumor cells (Fig. 1b). The optimal OS cutoff value for tumor CTLA-4 expression was 1.525 (sensitivity: 51.7 %; specificity: 67.3 %; area under curve [AUC]: 0.537; SE = 0.069) and for density of interstitial CTLA-4^+^ lymphocytes was 33.44/mm^2^ (sensitivity: 63.4 %; specificity: 69 %; AUC: 0.695; SE = 0.058) (ROC curves see Supplementary Figure 3).

### Prognostic factors for DFS and OS in breast cancer

Univariate analysis revealed that clinical outcome was significantly associated with established prognostic factors: age, clinical stage, Scarff-Bloom-Richardson (SBR) grade, tumor thrombus, estrogen receptor(ER) and progesterone receptor (PR) status, human epidermal growth factor receptor-2(HER2) expression and Ki67, but no significant association was found between clinical outcome and adjuvant chemotherapy (Supplementary Table 1).

### CTLA-4 expression and its correlation with clinicopathological features

No correlation between CTLA-4 expression in tumor cells and interstitial lymphocytes was found. Density of interstitial CTLA-4^+^ lymphocytes was not correlated with age, menopausal status, clinical stage, SBR grade, tumor thrombus, ER, PR, HER2 or Ki67. Tumor CTLA-4 expression was not correlated with age, clinical stage, SBR grade, tumor thrombus, ER, PR, HER2 and Ki67. Tumor CTLA-4^high^ expression was related to post-menstrual status (Mann–Whitney *U*, *P* = 0.008).

### Correlation between CTLA-4 expression and DFS or OS

Univariate analysis (log-rank) showed that lymphocyte density^high^ status was associated with longer DFS (*P* = 0.002) and OS (*P* = 0.004), but CTLA-4^high^ tumors with shorter OS (*P* = 0.041) (Table 2). Multivariate analysis found that (after controlling for age, clinical stage, SBR grade, tumor thrombus, ER, PR, HER2 and Ki-67) CTLA-4^+^ lymphocyte density^high^ was an independent predictor of longer DFS (HR 0.315, 95 % CL 0.150–0.658, *P* = 0.002) and OS (HR 0.313, 95 % CL 0.139–0.703, *P* = 0.005), whereas tumor CTLA-4^high^ was an independent predictor of shorter DFS (HR 2.176, 95 % CL 1.084–4.437, *P* = 0.029) and OS (HR 2.820, 95 % CL 1.337–5.950, *P* = 0.007). Clinical stage and HER-2 were also independent predictors of shorter DFS and OS.

**Table 2:** Univariate log-rank analysis of relationships between CTLA-4 expression and DFS, OS

Parameter	Basis for grouping	Number	DFS			OS		
			Recurrences	Mean DFS (month)	*P* value	Deaths	Mean OS (month)	*P* value
Density of interstitial CTLA-4^+^ lymphocytes	≤33.44/mm^2^	57	22	103.383	0.002	20	108.629	0.004
	>33.44/mm^2^	73	11	124.899		9	127.829	
Tumor CTLA-4 expression	≤1.525	82	17	126.029	0.088	14	124.771	0.041
	>1.525	48	16	103.607		15	110.287	

### Survival by CTLA-4 expression in tumor cells and density of primary tumor CTLA-4^+^ interstitial lymphocytes

Patients were divided into four subgroups according to profiles of CTLA-4 expression in tumor cells and density of CTLA-4^+^ interstitial lymphocytes: Group 1 (density^high^ CTLA-4^+^ lymphocytes, CTLA-4^low^ tumor cells), Group 2 (density^high^ CTLA-4^+^ lymphocytes, CTLA-4^high^ tumor cells), Group 3 (density^low^ CTLA-4^+^ lymphocytes, CTLA-4^low^ tumor cells) and Group 4 (density^low^ CTLA-4^+^ lymphocytes, CTLA-4^high^ tumor cells). Univariate analysis (log-rank) showed that DFS and OS were longer in Group 1 than in Group 2 (DFS: *P* = 0.006; OS: *P* = 0.008), Group 3 (DFS: *P* < 0.001; OS: *P* = 0.002) and Group 4 (*P* < 0.001 for both DFS and OS). Groups 2, 3 and 4 did not significantly differ in DFS or OS (*P* > 0.05) (Table 3).

**Table 3:** DFS and OS of groups divided by CTLA-4 expression profiles

Groups	Tumor CTLA-4 expression	Density of interstitial CTLA-4^+^ lymphocytes	Number	Recurrences	Mean DFS (month)	Deaths	Mean OS (month)
Group 1	≤1.525	>33.44/mm^2^	45	3	133.332	2	134.782
Group 2	>1.525	>33.44/mm^2^	28	8	107.649	7	114.033
Group 3	≤1.525	≤33.44/mm^2^	37	14	105.984	12	112.736
Group 4	>1.525	≤33.44/mm^2^	20	8	92.265	8	101.322

These results revealed that density^high^ interstitial CTLA-4^+^ lymphocytes to be a better prognosis factor only when tumor CTLA-4 expression was low (Group 1 vs. Group 3), whereas CTLA-4^high^ tumor cells were associated with worse prognosis only when density of interstitial CTLA-4^+^ lymphocytes was high (Group 1 vs. Group 2).

### Correlation between favorable CTLA-4 expression profile and prognosis

**Fig. 2:**
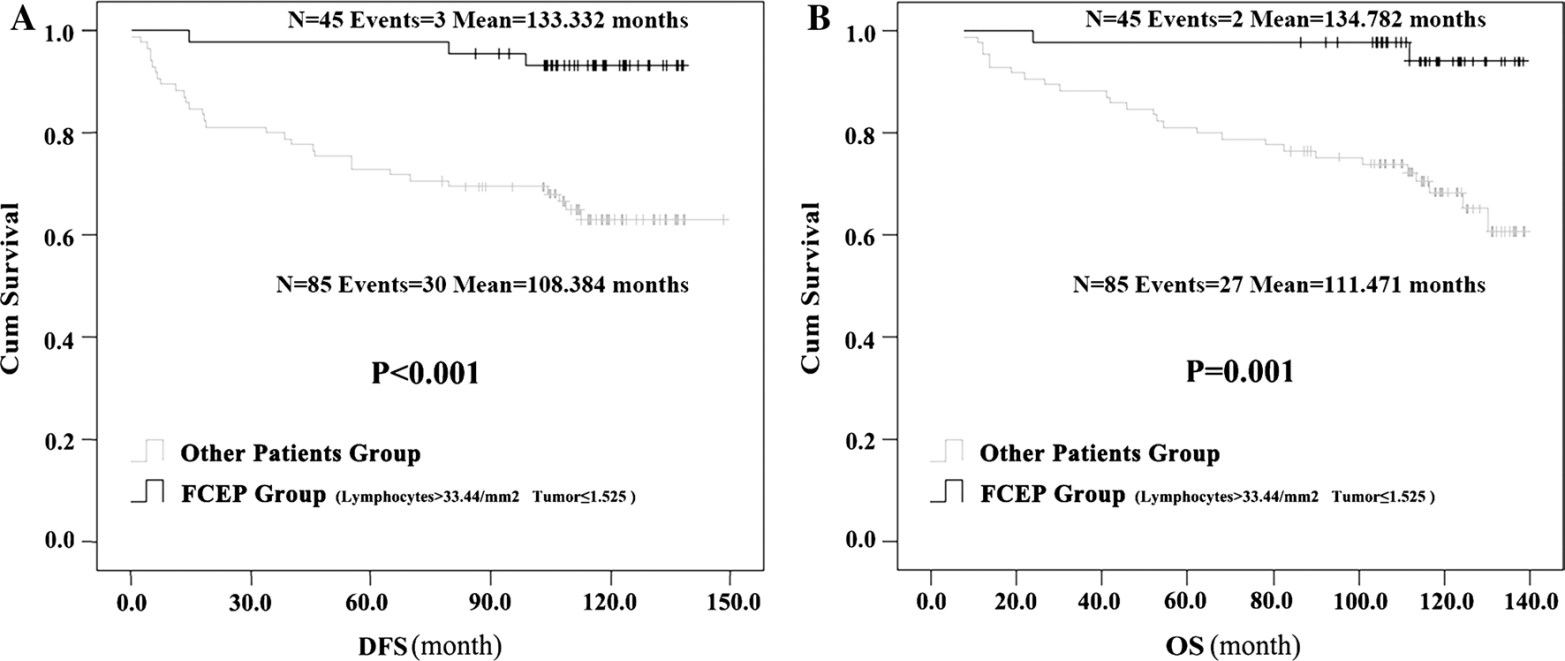
Kaplan–Meier survival curves on the correlation between favorable CTLA-4 expression profile and prognosis. Patients with high density CTLA-4^+^ interstitial lymphocytes (>33.44/mm^2^) and low CTLA-4 expression intensity in tumor cells (≤1.525) were characterized as the favorable CTLA-4 expression profile (FCEP) Group; the other patients were the Other Patients Group. Univariate analysis (log-rank) indicated that, the patients in FCEP group had longer DFS (**a**) and OS (**b**) than those of the Other Patients Group

Based on the aforementioned analysis, we renamed Group 1 as the “favorable CTLA-4 expression profile” (FCEP) Group, and patients in Groups 2, 3 and 4 collectively as the “Other Patients” Group. The HER-2^+^ rate was significantly lower in the FCEP Group than in Other Patients Group (12.50 vs. 34.92 %, *P* = 0.048, Pearson Chi-square test). But the two groups did not significantly differ in other clinical features. Univariate analysis (log-rank) showed the DFS of FCEP Group (*N* = 43, events = 3, mean DFS = 133.332 months) was longer than DFS of Other Patients Group(*N* = 85, events = 30, mean DFS = 108.384 months; *P* < 0.001), and OS of FCEP Group (*N* = 43, events = 2, mean OS = 134.782 months) was longer than OS of Other Patients Group(*N* = 85, events = 27, mean OS = 111.471 months; *P* = 0.001) (Fig. 2). Multivariate analysis showed that (after controlling for age, clinical stage, SBR grade, tumor thrombus, ER, PR, HER2 and Ki-67) FCEP status independently predicted longer DFS (HR 0.148, 95 % CL 0.045–0.489, *P* = 0.002) and OS (HR 0.116, 95 % CL 0.027–0.495, *P* = 0.004). Clinical stage, SBR grade and HER-2 were also independent predictors of DFS and OS.

As a predictor of good prognosis (survivals), the FCEP Group (density^high^ CTLA-4^+^ lymphocytes, CTLA-4^low^ tumor cells) was 42.6 % sensitive, 95.6 % specific and 53.85 % accurate, and its Youden index was 38.2 %.

## Discussion

### Elevated soluble CTLA-4 in tumor microenvironment associated with poor prognosis

The current study showed that CTLA-4 was expressed by tumor cells. CTLA-4^+^ dots in tumor cell cytoplasm looked like transport vesicles. Breast cancer cells reportedly also expressed CTLA-4, which was principally found in cytoplasm of cancer cells [10, 11]. Humans apparently produce both a full-length CTLA-4 isoform, found on cell membranes, and a soluble isoform of CTLA-4 (soluble CTLA-4) that only contains the extracellular domain [5]. An RT-PCR study of four breast cancer cell lines showed extracellular transcripts of CTLA-4 to be present in all four cell lines, but not the full-length form of CTLA-4 [11]. We therefore postulated that the CTLA-4^+^ dots in cytoplasm of breast cancer cells in the current study contained soluble CTLA-4, apparently synthesized by tumor cells and transported into the tumor microenvironment by transport vesicles.

Soluble CTLA-4 reportedly inhibits T-cell immunity, and CTLA-4 blockade could reverse this process [5, 8, 15]. One animal experiment suggested that soluble CTLA-4 plays a major role in immunosuppression, because selectively blocking soluble CTLA-4 alone by a specific antibody without blocking the full-length CTLA-4 isoform had an inhibitory effect on melanoma metastasis in vivo, similar to that attained by blocking both soluble and full-length isoforms of CTLA-4 [8]. Thus, CTLA-4^high^ expression in breast cancer cells might indicate an immunosuppressive tumor microenvironment, which could explain the association between higher CTLA-4 expression in breast cancer cells and poor prognosis, as demonstrated by this study.

Moreover, use of ipilimumab (CTLA-4 antibody) depleted CTLA-4^+^ melanoma cell lines through antibody-dependent cell-mediated cytotoxicity (ADCC), in vitro and in a mouse model [16], which implies that CTLA-4 blockade could both reverse immunosuppression in tumor microenvironment and destroy CTLA-4^+^ tumor cells directly through ADCC.

### Association between high density of CTLA-4^+^ lymphocytes and good prognosis

CTLA-4 expression is low on the surfaces of naive T cells and is up-regulated after T-cell activation to maintain immune self-tolerance and homeostasis [5, 8]. We therefore inferred that high density of CTLA-4^+^ lymphocytes indicates relatively strong immune function in the tumor microenvironment; in the present study, patients with density^high^ interstitial CTLA-4^+^ lymphocytes in their tumors understandably had better prognosis.

Anti-CTLA-4 blockade also depleted intratumoral CTLA-4^+^ Treg cells by macrophages via ADCC in recent reports [17–19], which implies that CTLA-4 blockade can activate anti-tumor immunity in the presence of enough tumor-infiltrating lymphocytes (TILs).

### Prognostic value of interstitial CTLA-4^+^ lymphocytes density was affected by tumor CTLA-4 expression

Although this study failed to associate tumor CTLA-4 expression with density of interstitial CTLA-4^+^ lymphocytes, the prognostic value of interstitial CTLA-4^+^ lymphocytes density was affected by CTLA-4 expression in tumor cells. Density^high^ patients had a survival advantage only if tumor CTLA-4 expression was low. Based on the aforementioned analysis, we believed that CTLA-4^high^ tumor cells indicated high levels of soluble CTLA-4 in tumor microenvironment, which suppressed the function of lymphocytes that were activated when infiltrating tumors.

For patients of Group 2 (CTLA-4^high^ tumor cells, density^high^ CTLA-4^+^ lymphocytes), high levels of soluble CTLA-4 in the tumor microenvironment would suppress TIL anti-tumor immunity to no stronger than that of the density^low^ groups, which would lead to poor prognosis. As Group 1/FCEP Group had the most favorable immunological status (CTLA-4^low^ tumor cells, density^high^ CTLA-4^+^ lymphocytes, indicative of active immune reaction), their superior prognosis among all patients made sense.

### Implication of CTLA-4 expression profile for individualized immunotherapy

As a negative regulator of T-cell immunity, CTLA-4 is an attractive target for cancer immunotherapy. Altering the balance between CD28/B7 positive and CTLA-4/B7 negative regulatory signals may enhance anti-tumor immune response. However, remaining questions include (a) how far this balance can be shifted to be effective while avoiding serious immunity-related adverse reactions and (b) how to monitor such a change in immune balance. In clinical trial, although only a subset of melanoma patients treated with anti-CTLA-4 antibody derived benefits, immunity-related adverse events of different severities occurred in approximately 64.2 % of melanoma patients treated with anti-CTLA-4 antibody, some of which were lethal [20, 21].

A clinical trial of CTLA-4 blockade treatment for breast cancer showed that peripheral blood immune status was improved after CTLA-4 blockade in most patients, but good peripheral blood immune responses did not invariably translate into lasting beneficial outcomes [22]. This phenomenon suggests that the immunosuppressive state of the tumor microenvironment is more potent and complicated than that in peripheral blood and, therefore, much more difficult to be overcome. An effective cancer immunotherapy should be able to induce effective anti-tumor immunity and, at the same time, remove tumor-induced immunosuppression in its microenvironment.

In this study, breast cancer patients were divided into four groups by their CTLA-4 expression profiles in tumor cells and primary TILs. These profiles could facilitate tailoring immunotherapeutic strategies to patients’ different immunological features.

Group 2 patients(CTLA-4^high^ tumor cells, density^high^ CTLA-4^+^ lymphocytes) might benefit most from CTLA-4 blockade treatment, because a sufficient number of preexisting inactive T cells in tumor microenvironment could be re-activated after neutralizing soluble CTLA-4 with CTLA-4 antibodies; ADCC against CTLA-4^+^ tumor cells might also be induced by CTLA-4 antibodies. For Group 4 patients (CTLA-4^high^ tumor cells, density^low^ CTLA-4^+^ lymphocytes), CTLA-4 blockade alone would not induce an effective anti-tumor immunity given the paucity of preexisting TILs; combination therapies aimed at eliciting anti-tumor immunity are needed. CTLA-4 blockade might not benefit patients in Groups 1 and 3 (those with CTLA-4^low^ tumor cells) because of the scant levels of soluble CTLA-4 to neutralize. Moreover, given an adequate number of preexisting active T cells in the Group 1 tumor microenvironment, the balance between CD28/B7 positive and CTLA-4/B7 negative regulatory signals may be overthrown by CTLA-4 blockade, thus evoking excessive immune response and immunity-related toxicity.

## Conclusion

We found CTLA-4 expression in primary breast cancer lesions to have potential prognostic value; higher CTLA-4 expression in breast cancer cells was associated with worse prognosis, and higher density of interstitial CTLA-4^+^ lymphocytes with better prognosis. However, high CTLA-4^+^ lymphocyte density was significantly correlated with good prognosis only when tumor CTLA-4 expression was low. CTLA-4 expression varied greatly among breast cancer patients; we identified CTLA-4 expression profiles in terms of CTLA-4 expression in lymphocytes and tumor cells. We speculated that these immunological features might be associated with clinical efficacy and adverse reactions to CTLA-4 blockade treatment and should help to guide immunotherapeutic strategies. Further studies of immunotherapies guided by individual variation in immune status are warranted.

### Electronic supplementary material

Below is the link to the electronic supplementary material.

Supplementary material 1 (PDF 126 kb)

## Abbreviations

ADCC: Antibody-dependent cell-mediated cytotoxicity
cSMAC: Central supramolecular activation cluster
CTLA-4: Cytotoxic T lymphocyte antigen 4
DFS: Disease-free survival
ER: Estrogen receptor
FCEP: Favorable CTLA-4 expression profile
HER2: Human epidermal growth factor receptor-2
OS: Overall survival
PBMC: Peripheral blood mononuclear cell
PR: Progesterone receptor
ROC analysis: Receiver-operating characteristic analysis
SBR grade: Scarff-Bloom-Richardson grade
Tregs: Regulatory T cells
