# Supplementary material for: Cytotoxic T lymphocyte antigen 4 expression in human breast cancer: implications for prognosis
Source: Cancer Immunol Immunother. 2015 Apr 17;64(7):853–60. doi: 10.1007/s00262-015-1696-2 (PMC4481300; doi:10.1007/s00262-015-1696-2)
Supplement: Supplementary file 1 — Supplementary material 1 (PDF 126 kb) [file 262_2015_1696_MOESM1_ESM.pdf]

Supplementary table 1. Prognostic factors for overall survival (OS) and disease-free survival (DFS) in univariate cox analysis.

Influencing factors	DFS		OS	
	HR (95%CL)	P value	HR(95%CL)	P value
Clinical stage	2.599(1.509-4.478)	0.001	3.156(1.736-5.738)	<0.001
Age	0.489(0.237-1.009)	0.053	0.673(0.317-1.427)	0.301
Menstrual status	0.995(0.489-2.023)	0.988	1.315(0.627-2.755)	0.469
ER	0.568(0.286-1.128)	0.106	0.422(0.203-0.879)	0.018
PR	0.473(0.237-0.944)	0.034	0.368(0.173-0.782)	0.009
HER-2	3.977(1.990-7.947)	<0.001	5.398(2.601-11.200)	<0.001
Thrombosis	2.680(1.332-5.395)	0.006	2.993(1.420-6.310)	0.004
SBR grading	3.204(1.712-5.997)	<0.001	3.904(1.982-7.687)	<0.001
Ki67	2.119(1.042-4.311)	0.038	2.556(1.163-5.617)	0.019
Adjuvant chemotherapy	1.115(0.460-2.702)	0.809	0.923(0.376-2.270)	0.862

Cancer Immunology, Immunotherapy (submitted in 2014) - Haiming Yu et al.

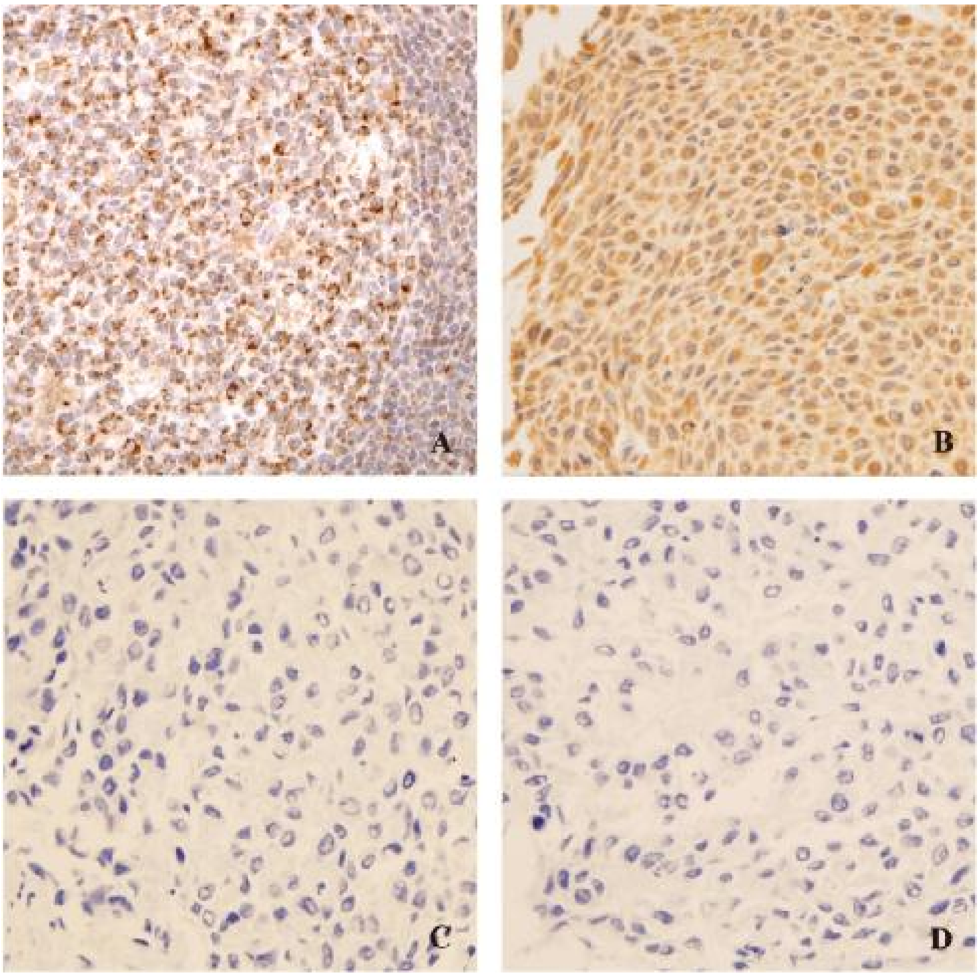

Supplementary figure1 Positive control and negative controls for rabbit anti-human CTLA-4 IgG. **(A)** Sections of human tonsil biopsy specimens with confirmed high expression of CTLA-4 molecules served as **positive control**; **(B)** Positive immunostaining for CTLA-4 in breast cancer cells; **(C)** Sections of breast cancer specimens incubated with the primary antibody diluent with no primary antibody were used as the **negative control 1**; **(D)** Sections of breast cancer specimens incubated with primary antibody (1:100 dilution, bs-1179R, Beijing Biosynthesis Biotechnology Co., Ltd, China) premixed with CTLA-4 peptide (bs-1179P, Beijing Biosynthesis Biotechnology Co., Ltd, China) were used as the **negative control 2**.

Cancer immunology, immunotherapy (submitted in 2014) – Haiming Yu et al.

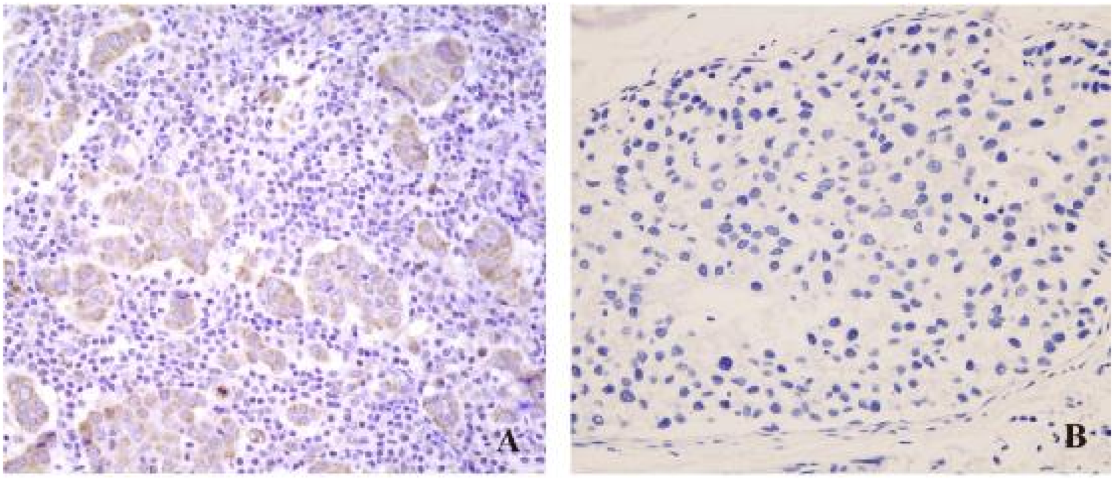

Supplementary figure2 Examples for CTLA-4 staining intensities. (A) Tumor cell staining intensity score 1. **(B)** Tumor cell staining intensity score 0.

Cancer immunology, immunotherapy (submitted in 2014) – Haiming Yu et al.

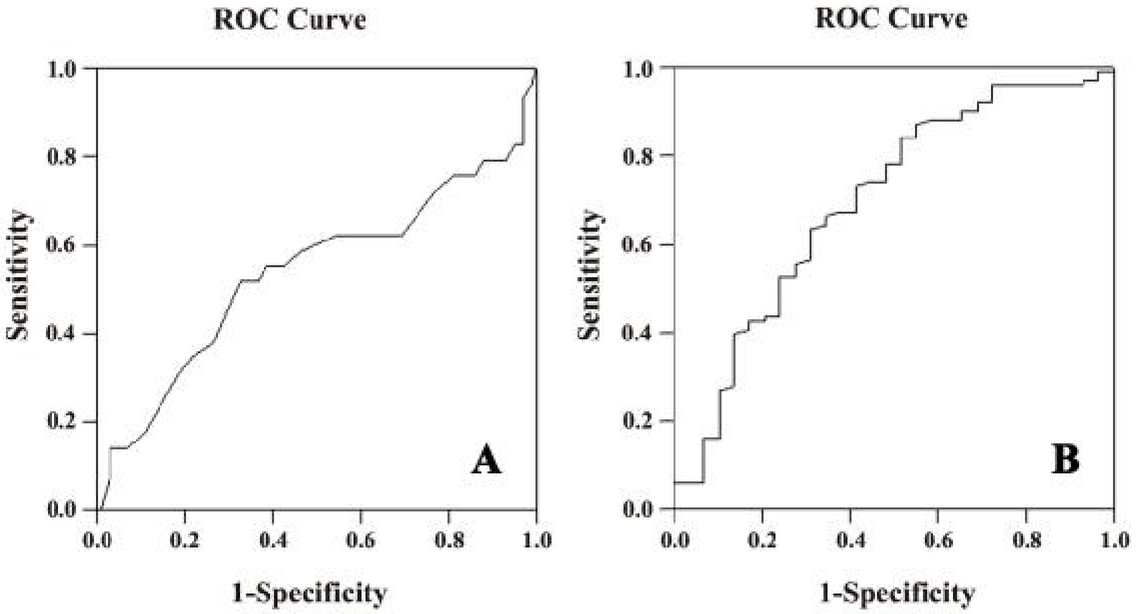

Supplementary figure 3 Receiver operator characteristic (ROC) curves of CTLA-4 expression for overall survival in breast cancer patients. (A) Tumor CTLA-4 expression: sensitivity=51.7%; specificity=67.3%; area under the curve=0.537; SE=0.069; **(B)** Density of interstitial CTLA-4^{+} lymphocytes: sensitivity=63.4%; specificity=69%; area under the curve=0.695; SE=0.058.

Cancer Immunology, Immunotherapy (submitted in 2014) - Haiming Yu et al.
